# Does Pneumatic Tube System Transport Contribute to Hemolysis in ED Blood Samples?

**DOI:** 10.5811/westjem.2016.6.29948

**Published:** 2016-07-26

**Authors:** Michael P. Phelan, Edmunds Z. Reineks, Fredric M. Hustey, Jacob P. Berriochoa, Seth R. Podolsky, Stephen Meldon, Jesse D. Schold, Janelle Chamberlin, Gary W. Procop

**Affiliations:** * Cleveland Clinic Health Systems, Emergency Services Institute, Cleveland, Ohio; † Cleveland Clinic Health Systems, Pathology and Laboratory Medicine Institute, Cleveland, Ohio; ‡ Cleveland Clinic Health Systems, Quantitative Health Sciences, Cleveland, Ohio; § MetroHealth Medical Center, Emergency Medicine/Emergency Department, Cleveland, Ohio

## Abstract

**Introduction:**

Our goal was to determine if the hemolysis among blood samples obtained in an emergency department and then sent to the laboratory in a pneumatic tube system was different from those in samples that were hand-carried.

**Methods:**

The hemolysis index is measured on all samples submitted for potassium analysis. We queried our hospital laboratory database system (SunQuest^®^) for potassium results for specimens obtained between January 2014 and July 2014. From facility maintenance records, we identified periods of system downtime, during which specimens were hand-carried to the laboratory.

**Results:**

During the study period, 15,851 blood specimens were transported via our pneumatic tube system and 92 samples were hand delivered. The proportions of hemolyzed specimens in the two groups were not significantly different (13.6% vs. 13.1% [p=0.90]). Results were consistent when the criterion was limited to gross (3.3% vs 3.3% [p=0.99]) or mild (10.3% vs 9.8% [p=0.88]) hemolysis. The hemolysis rate showed minimal variation during the study period (12.6%–14.6%).

**Conclusion:**

We found no statistical difference in the percentages of hemolyzed specimens transported by a pneumatic tube system or hand delivered to the laboratory. Certain features of pneumatic tube systems might contribute to hemolysis (e.g., speed, distance, packing material). Since each system is unique in design, we encourage medical facilities to consider whether their method of transport might contribute to hemolysis in samples obtained in the emergency department.

## INTRODUCTION

Emergency department (ED) blood samples have a high rate of hemolysis (6%–30%) when sent to the clinical laboratory for analysis.^1^–^3^ ED hemolysis is much higher than the 2% benchmark established by the American Society for Clinical Pathology. This is especially problematic given the high-volume, crowded nature of an ED.^4^ Hemolysis of a sample often requires repeat specimens to be drawn and tested. These repeat tests delay treatment and clinical decision making, prolong patients’ ED length of stay, and cause patient dissatisfaction due to multiple sticks for repeated blood draws. While the reasons for high hemolysis rates are likely multifactorial, they are typically caused by the pre-analytic phase of the testing process.^2^ One possible source is the use of pneumatic tube transport systems to transfer the test tubes from the ED to the clinical laboratory.^5^,^6^

Pneumatic tube transport systems are common in modern hospitals because they increase the speed of sample delivery to the laboratory. They have been shown to decrease laboratory turnaround time,^7^–^9^ which is a benchmark quality indicator in “stat” laboratory testing.^10^ However, the decrease in turnaround time might come at the cost of sample quality. Ellis and Hasan both led ED-based studies that revealed significantly increased specimen hemolysis rates after pneumatic tube systems were installed.^5^,^6^ In contrast, other studies of pneumatic tube use demonstrated no significant changes in hemolysis rates.^8^,^9^,^11^–^15^ Because of this discrepancy in the literature, and in search of techniques to lower our own institution’s hemolysis rates, we sought to determine whether hemolysis rates of blood samples obtained in the ED were increased when transported in a pneumatic tube system versus being hand carried to the lab.

## METHODS

### Study Design

This work was part of a larger performance improvement program aimed at reducing rates of hemolysis in blood specimens obtained in an urban, tertiary referral ED with an annual census of 64,000. The hospital laboratory database system (SunQuest^®^) was queried for potassium results for specimens obtained during a seven-month study period (January–July 2014). Although other analytes can be affected by hemolysis, we chose to analyze hemolysis in potassium due to the frequency and patient impact of a hemolyzed potassium sample. From facility maintenance records, we found discrete time periods lasting 60 minutes or more (eventually when added totaling a cumulative of 24 hours) when specimens were hand carried due to pneumatic tube system downtime, as logged by the laboratory’s central specimen receiving desk. These discrete downtime periods were used because they were considered to be the most valid comparison. We determined the proportions of samples with hemolysis that were hand-carried and transported by the pneumatic tube system. Pneumatic tube transport involved sending samples through the system to a building across the street with an estimated distance of no more than 500 feet point to point. There are numerous bends to accommodate launch and receiving locations within this process. Because this was part of a quality improvement project this study was granted exemption from institutional board review approval.

### Sample Analysis

The hemolysis index is measured on all plasma and serum samples submitted for potassium analysis. A dimensionless hemolysis index is available from our automated instrumentation (Roche cobas8000, c702 analyzer), which provides the index as a quality indicator. If hemolysis is detected, the result is reported in the electronic medical record as either grossly hemolyzed and rejected (GHEMO) or mildly hemolyzed (HK), with a cautionary comment added to the numerical result.

### Statistical Analysis

We performed two-sided chi-squared tests with a type I error of 0.05 with SAS^®^ (v.9.2., Cary, NC).

## RESULTS

During the seven-month study period, 15,851 blood specimens were collected in the ED and transported to the laboratory using standard methodology with the pneumatic tube system. Ninety-two samples were hand delivered during the system’s downtime. The proportions of combined hemolyzed specimens in the pneumatic tube (13.6%) and hand-carried (13.1%) groups (GHEMO or HK) were not significantly different (p=0.90, Figure). Results were consistent when the hemolysis criterion was limited to GHEMO (3.3% vs 3.3% [p=0.99]) alone or HK (10.3% vs 9.8% [p=0.88]) alone. Overall hemolysis rates showed minimal variation during the study period (12.6%–14.6%).

## DISCUSSION

This study showed no statistical difference in the percentages of hemolyzed specimens among those transported by a pneumatic tube system or hand delivered from our ED. Our findings are similar to those of Fernandes et al and Stair et al, who also found no statistical difference in hemolysis rates between the two methods of transport of samples from their EDs.^8^,^15^ In contrast, Ellis, Hasan, and Steige each found a large increase in hemolysis with the implementation of pneumatic tube systems in their institutions.^5^,^6^,^16^

Each pneumatic tube system is unique. Their characteristics (e.g., delivery route, velocity) appear to affect sample pressures during transport, which might influence hemolysis rates. Variables that have been shown to cause cell deterioration and increase hemolysis include rapid accelerations and decelerations,^17^,^18^ increased length of travel, and high speeds.^13^,^18^ Other variables that reduce hemolysis during transport include the use of gel tubes,^19^ properly filled tubes,^16^ and padded inserts within the canisters.^12^

In our facility, hemolysis in non-ED samples occurs at about an incidence of 3%, in contrast to the higher incidence observed in our ED samples (13.5%). ED samples are routinely obtained during intravenous (IV) placement while the standard process for obtaining inpatient and ambulatory samples is via straight-stick method, which is known to have a lower hemolysis rate. 1, 2

## LIMITATIONS

The study is a retrospective chart review and therefore may carry some limitations typically associated with such reviews. It is possible that some hand-carried specimens were included in the pneumatic tube sample, as hand-carried specimens were only identified for downtime periods of 60 minutes or greater when the pneumatic tube system was not functioning. If significant numbers of hand-carried samples were inadvertently included in the pneumatic tube group, this may have in part contributed to the finding of no difference between the two groups. However, the large sample size of the pneumatic tube group should have mitigated any such effect. In addition, we chose the discrete downtime periods for hand-transported samples in order to minimize the possibility of inadvertent inclusion of pneumatic tube samples in this group. Relying on engineering logs to identify downtimes could have resulted in missing some time periods when the pneumatic tube system was down but not recorded.

While it was standard practice in the ED during the study period to obtain samples during IV placement, there may be heterogeneity in phlebotomy technique, equipment and the experience of the phlebotomist, which can be a confounding factor affecting the data. This analysis was performed at an early phase of our larger performance improvement project. However, the study period occurred prior to implementation of any process improvement activities aimed at reducing hemolysis. This study was performed in a single ED with unique characteristics (pneumatic tube system, blood drawing techniques) and may not be generalizable to other settings.

## CONCLUSION

Our pneumatic tube system does not increase the rate of hemolysis in blood samples collected in our ED. We encourage each institution to examine the features of its pneumatic tube system and consider whether its characteristics and configuration might be contributing to the hemolysis of blood samples.

## Figures and Tables

**Figure:**
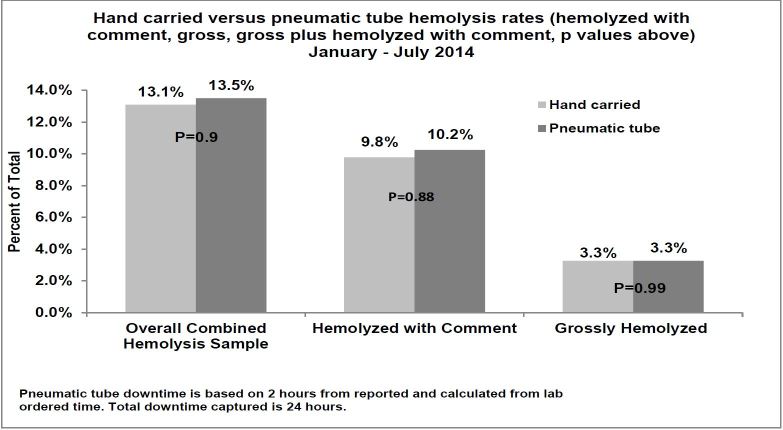
Comparison of hand carried vs pneumatic tube transported emergency department sample hemolysis January – July 2014.
